# Primary Dural Diffuse Large B-Cell Lymphoma: A Case Report

**DOI:** 10.31662/jmaj.2025-0198

**Published:** 2025-09-05

**Authors:** Yoshiaki Takamura, Takatoshi Fujimoto

**Affiliations:** 1 Department of Neurosurgery, Higashiosaka City Medical Center, Osaka, Japan

**Keywords:** primary central nervous system lymphoma, dura mater, diffuse large B-cell lymphoma

## Abstract

Primary dural lymphoma is a rare subtype of primary central nervous system lymphoma that arises from the dura mater. The majority of primary dural lymphomas are histologically mucosa-associated lymphoid tissue lymphoma. We present a rare case of a 78-year-old woman with primary dural lymphoma diagnosed as diffuse large B-cell lymphoma. She was admitted with status epilepticus. Magnetic resonance imaging showed an extra-axial mass lesion in the right parieto-occipital region, which enhanced homogeneously with contrast medium. To obtain a histopathological diagnosis, resection of the lesion involving the dura mater was performed. Based on histopathological and immunohistochemical examinations, the patient was diagnosed with diffuse large B-cell lymphoma. We summarize the clinical characteristics of this unusual disease.

## Introduction

Primary dural lymphoma (PDL) is a rare subtype of primary central nervous system lymphoma (PCNSL) that arises from the dura mater. The majority of PDLs are histologically mucosa-associated lymphoid tissue (MALT) lymphomas. We present a rare case of a 78-year-old woman with PDL diagnosed as diffuse large B-cell lymphoma (DLBCL).

## Case Report

**Figure 1.**
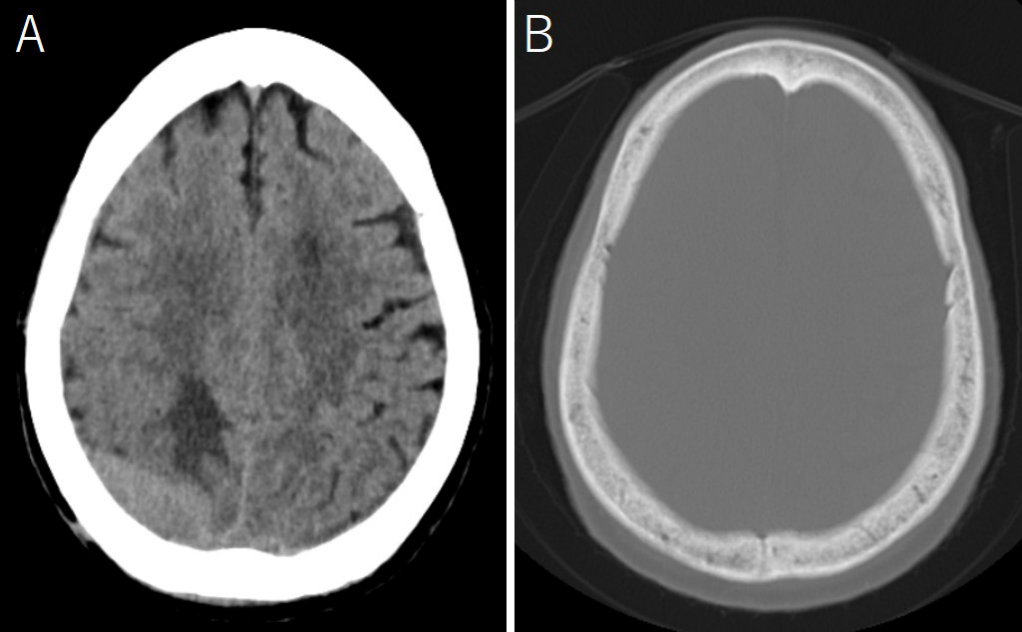
CT showed a slightly high-density mass in the right parieto-occipital region with peritumoral edema (A). The bone window view showed no signs of invasion by the tumor (B). CT: computed tomography.

**Figure 2.**
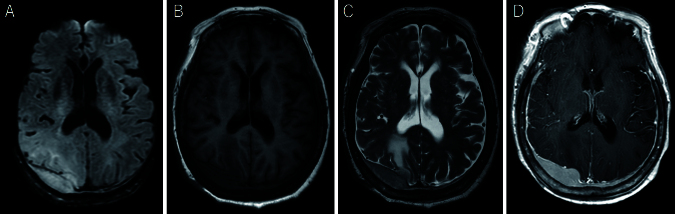
MRI. Dural-based extra-axial mass lesion showed slight hyperintensity in diffusion-weighted image (A), isointensity in T1-weighted image (B), and slight hyperintensity in T2-weighted image (C). Post-contrast scans showed homogeneous enhancement of the mass with a long dural tail sign (D). MRI: magnetic resonance imaging.

**Figure 3.**
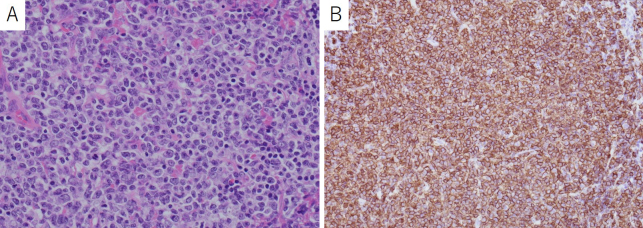
The tumor is histologically composed of large lymphoid cells with a predominantly diffuse growth pattern (HE stain) (A). The tumor cells are immunoreactive for CD20 (B). HE: hematoxylin and eosin.

A 78-year-old immunocompetent woman was admitted with a generalized tonic-clonic seizure. A computed tomography (CT) scan showed a slightly high-density mass in the right parieto-occipital region with peritumoral edema (Figure 1A). The bone window view showed no signs of tumor invasion (Figure 1B). On magnetic resonance imaging (MRI), the dural-based extra-axial mass lesion showed slight hyperintensity on diffusion-weighted image, isointensity on T1-weighted image, and slight hyperintensity on T2-weighted image (Figure 2A-C). Post-contrast scans showed homogeneous enhancement of the mass with a long dural tail sign (Figure 2D). Serum immunology studies and cerebrospinal fluid examination were unremarkable. There were no additional lesions on CT of the chest and abdomen. Based on these findings, the differential diagnosis included meningioma, solitary fibrous tumor, metastatic tumors of unknown origin, and lymphoma. To obtain a histopathological diagnosis, subtotal resection was performed, except for the area of extensive dural involvement. There was no adhesion to the brain. Microscopic examination showed large lymphoid cells with a predominantly diffuse growth pattern (Figure 3A). Immunochemical studies showed positive CD20 (Figure 3B). CD79a+, CD10+, BCL6+, MUM1−, BCL2−, and MYC+ were also observed. Ki67 staining showed a proliferation fraction of 60%. Based on these findings, the patient was diagnosed with DLBCL arising from the dura mater. The patient was transferred to the other institute with a hematology department for chemotherapy.

## Discussion

PCNSL is a malignant type of non-Hodgkin lymphoma that is observed only in the central nervous system. PCNSL most commonly arises from the brain parenchyma. PDL is a rare subtype of PCNSL that arises from the dura mater, and accounts for 2·4%-6·3% of all PCNSL ^(1), (2)^. Fattahi et al. ^(3)^ reviewed reported PDL cases in the literature. PDL occurs more often in middle-aged women, in contrast to parenchyma PCNSL, which predominantly affects high-aged men. This lesion usually presents with headaches, seizures, cranial nerve deficits, and focal neurological deficits. The cerebral convexities are the most common site, but other sites include the falx, tentorium, sellar, and sphenoid wing. Parenchyma PCNSL is commonly classified as DLBCL, whereas the majority of PDLs are histologically MALT lymphoma. Primary dural DLBCL, as seen in the present case, is extremely rare. The pathogenesis of PDL is still unknown because the dura lacks lymphoid tissue. One hypothesis for its development involves the presence of meningothelial cells throughout the arachnoid membrane. Meningothelial cells are analogous to epithelioid cells at other sites where MALT lymphoma arises ^(4)^. This suggests that MALT lymphoma constitutes the majority of PDL.

The neuroimaging findings of PDL are similar to meningioma ^(1), (2), (3)^. PDL presents with extra-axial lesions that diffusely enhance with gadolinium administration. Furthermore, a dural tail is often observed. Thus, PDL may be misdiagnosed as meningioma. Menniti et al. ^(5)^ reported that among 14 previously documented cases of PDL, 13 were initially diagnosed as meningiomas. Similarly, in another report, 14 out of 15 patients with PDL were radiographically diagnosed with meningioma ^(6)^. The presence of vasogenic edema and parenchymal brain invasion with a fuzzy tumor-brain interface suggests PDL ^(7)^. Karschnia et al. ^(2)^ described the MRI characteristics of PDL on diffusion-weighted imaging sequences and apparent diffusion coefficient maps. Among 15 cases, diffusion-weighted imaging intensity was predominantly hyperintense compared to white matter, and the apparent diffusion coefficient value was markedly lower than that of meningiomas. When radiographic examination does not yield a definitive diagnosis, pathological examination, following craniotomy or directed biopsy, is the only method to confirm the diagnosis.

There are few reports about PDL, and its standard treatment remains unclear. Because complete resection may be difficult due to extensive invasion, adjuvant treatment is necessary in most cases ^(3)^. MALT lymphoma responds favorably to radiotherapy due to its high radiosensitivity; a five-year survival rate of 86% has been reported ^(1)^. On the other hand, chemotherapy is mostly selected for dural DLBCL ^(3)^. Chemotherapy might be effective because the drugs do not need to cross the blood-brain barrier. The prognosis of dural DLBCL appears to be favorable than that of parenchymal DLBCL; the average survival time of patients with dural DLBCL was 29.3 months ^(8)^. However, an optimal treatment remains under debate due to the rarity of the disease. In the future, more cases and long-term follow-up data need to be accumulated.

One limitation of this case report is the lack of a bone marrow biopsy. Thus, there is a possibility that the dural lesion could be a manifestation of systemic lymphoma.

## Article Information

### Author Contributions

The authors contributed to the study conception and design, performed data collection, made substantial contributions to the analyses and interpretation of the data, and wrote this manuscript.

### Conflicts of Interest

None

### Informed Consent

The patient had agreed to publish the case in an academic journal without exposing his identity.

### IRB Approval Code and Name of the Institution

Not applicable

## References

[1] Iwamoto FM, Abrey LE. Primary dural lymphomas: a review. Neurosurg Focus. 2006;21(5):E5. 10.3171/foc.2006.21.5.6 17134121

[2] Karschnia P, Batchelor TT, Jordan JT, et al. Primary dural lymphomas: clinical presentation, management, and outcome. Cancer. 2020;126(12):2811-20. 32176324 10.1002/cncr.32834

[3] Fattahi A, Sadeghipour A, Shayanfar N, et al. Primary dural lymphoma: a comprehensive literature review and report of a case. Oncol Clin Pract. 2022;18(5):335-48.

[4] Ferguson SD, Musleh W, Gurbuxani S, et al. Intracranial mucosa-associated lymphoid tissue (MALT) lymphoma. J Clin Neurosci. 2010;17(5):666-9. 20202849 10.1016/j.jocn.2009.10.001

[5] Menniti A, Moschettoni L, Liccardo G, et al. Low-grade primary meningeal lymphoma: case report and review of the literature. Neurosurg Rev. 2005;28(3):229-33. 15682333 10.1007/s10143-004-0373-2

[6] Tu PH, Giannini C, Judkins AR, et al. Clinicopathologic and genetic profile of intracranial marginal zone lymphoma: a primary low-grade CNS lymphoma that mimics meningioma. J Clin Oncol. 2005;23(24):5718-27. 16009945 10.1200/JCO.2005.17.624

[7] Iwamoto FM, DeAngelis LM, Abrey LE. Primary dural lymphomas: a clinicopathologic study of treatment and outcome in eight patients. Neurology. 2006;66(11):1763-5. 16769960 10.1212/01.wnl.0000218284.23872.eb

[8] Yamada SM, Ikawa N, Toyonaga S, et al. Primary malignant B-cell-type dural lymphoma: case report. Surg Neurol. 2006;66(5):539-43. 17084207 10.1016/j.surneu.2006.02.033

